# The preference–performance relationship as a means of classifying parasitoids according to their specialization degree

**DOI:** 10.1111/eva.12822

**Published:** 2019-06-17

**Authors:** Lucie S. Monticelli, Le Thu Ha Nguyen, Edwige Amiens‐Desneux, Chen Luo, Anne‐violette Lavoir, Jean‐Luc Gatti, Nicolas Desneux

**Affiliations:** ^1^ Université Côte d'Azur, INRA, CNRS UMR ISA Nice France

**Keywords:** aphid, generalist parasitoids, host range, preference–performance hypothesis, specialization

## Abstract

Host range in parasitoids could be described by the preference–performance hypothesis (PPH) where preference is defined as host acceptance and performance is defined as the sum of all species on which parasitoid offspring can complete their life cycle. The PPH predicts that highly suitable hosts will be preferred by ovipositing females. However, generalist parasitoids may not conform to this hypothesis if they attack a large range of hosts of varying suitability. Under laboratory conditions, we tested the PPH relationship of three aphid parasitoids currently considered as generalist species (*Aphelinus abdominalis, Aphidius ervi, Diaeretiella rapae*). As expected, the three parasitoids species showed low selectivity, i.e., females stung all aphid species encountered (at least in some extent). However, depending on the parasitoid species, only 42%–58% of aphid species enabled producing parasitoid offspring. We did not find a correlation between the extent of preference and the performance of three generalist aphid parasitoids. For *A. ervi*, host phylogeny is also important as females showed higher attack and developmental rates on hosts closely related to the most suitable one. In addition, traits such as (a) the presence of protective secondary endosymbionts, for example, *Hamiltonella defensa* detected in *Aphis fabae* and *Metopolophium dirhodum* and (b) the sequestration of plant toxins as defense mechanism against parasitism, for example, in *Aphis nerii* and *Brevicoryne brassicae*, were likely at play to some extent in narrowing parasitoid host range. The lack of PPH relationship involved a low selectivity leading to a high adaptability, as well as selection pressure; the combination of which enabled the production of offspring in a new host species or a new environment. Testing for PPH relationships in parasitoids may provide useful cues to classify parasitoids in terms of specialization degree.

## 1 INTRODUCTION

Host specificity, and more broadly diet breath, has been described by the preference–performance hypothesis (PPH) of Jaenike (1978). It predicts a positive relationship between the choice of adult females (preference) and the degree of successful offspring development (performance). Various studies have shown support for the preference–performance hypothesis for specialized phytophagous arthropods (Craig, Itami, & Price, 1989; Gripenberg, Mayhew, Parnell, & Roslin, 2010; Jaenike, 1978; Nylin & Janz, 1993; Thompson, 1988) as well as for specialized predators (Sadeghi & Gilbert, 1999) and parasitoids (Brodeur, Geervliet, & Vet, 1998; Desneux, Barta, Hoelmer, Hopper, & Heimpel, 2009; Driessen et al., 1991). However, it has been argued that such relationships may not be common in the case of generalist arthropods (Chesnais, Ameline, Doury, Roux, & Couty, 2015; Eben, Benrey, Sivinski, & Aluja, 2000; Gripenberg et al., 2010).

Parasitoids are insects of which free‐living adult females deposit eggs in, on, or near hosts and immature stages develop by host consumption (parasitic stage; Godfray, 1994). Hence, the host represents the only food source for the parasitoid larvae and female choice to lay its egg is decisive. The selection of hosts by parasitoids involves the detection of physical and/or chemical cues from the other trophic levels (e.g., host species and/or host plants) (Mackauer, Michaud, & Völkl, 1996; Vet & Dicke, 1992; Vinson, 1985), and the host specificity of parasitoids may be mainly shaped by infochemicals, i.e., chemicals emitted by host and/or host plant (Afsheen, Wang, Li, Zhu, & Lou, 2008). Specialist parasitoids may use more specific cues related to their hosts (Barbosa, 1988; McCormick, Unsicker, & Gershenzon, 2012; Vet & Dicke, 1992). This is the case, for example, for *Microplitis croceipes* that uses host kairomones from a variety of host‐related sources (e.g., frass, hemolymph, and salivary secretions; Alborn, Lewis, & Tumlinson, 1995; Jones, Lewis, Bowman, Beroza, & Bierl, 1971). By contrast, generalist parasitoids often use more generalized cues to identify potential host species (Vet & Dicke, 1992). For example, the generalist fly parasitoid *Aphaereta minuta* does not use host‐derived chemical cues to select host larvae and attacks almost all hosts that are present in encountered decaying materials (Vet, 1985).

Assessing PPH relationships in parasitoids may provide useful clues to classify parasitoids in terms of specialization degree; i.e., specialized parasitoids may show significant PPH relationships, whereas generalist ones rarely do. However, several studies have reported positive preference–performance relationships in parasitoids considered to be generalists (Kos et al., 2012; Li, Miller, & Sun, 2009). It should be noted though that all of these studies (a) tested only a few host species (a maximum of 3), and (b) the host species belonged to the same tribes or genus thus representing a possible bias in assessing the preference–performance correlation (Poulin & Mouillot in, 2005). Such reported positive PPH relationships may actually be false positives in the sense that these studies were not designed per se to assess the link between the preference of females and the performance of offspring in the context of the PPH (Gripenberg et al., 2010).

In this context, we assessed the PPH relationship in three aphid parasitoid species *Aphelinus abdominalis* (Aphelinidae), *Aphidius ervi* (Braconidae), and *Diaeretiella rapae* (Braconidae) that have been considered generalists, i.e., attacking a broad phylogenetic range of aphids (Honek, Jarosik, Lapchin, & Rabasse, 1998; Kavallieratos et al., 2004), through characterization of the behavioral (preference) and physiological (performance) determinants of host specificity of these parasitoid species under laboratory conditions. To achieve this, we used twelve aphid species that feed on six different host plants and spread over two different tribes (Aphidini and Macrosiphini) within the subfamily Aphidinae. They were chosen to cover a broad phylogenetic range of aphid species (Coeur d'Acier, Jousselin, Martin, & Rasplus, 2007; Desneux, Barta, Hoelmer, et al., 2009; von Dohlen, Rowe, & Heie, 2006). In addition, we calculated the host specificity index $S_{\text{TD}}^{\text{*}}$ from Poulin and Mouillot (2005) to classify the parasitoids according to their host specificity; doing so we identified an endpoint for quantifying where these species lie on a generalist–specialist continuum. For this, we considered also previous results on specialist parasitoids generated by our laboratory (Desneux, Barta, Hoelmer, et al., 2009; Monticelli, 2018). Finally, because various ecological factors such as secondary endosymbionts (Hopkinson, Zalucki, & Murray, 2013; Oliver, Russell, Moran, & Hunter, 2003) may modulate the preference and/or performance of parasitoids (Monticelli, Outreman, Frago, & Desneux, 2019), each aphid colony was screened for the presence of nine secondary endosymbionts.

## 2 MATERIALS AND METHODS

### 2.1 Biological materials

All the aphid colonies used in the study were initiated from individuals collected in France, and all colonies were mixtures of clones. The description of the aphid species, their color, hosts plants, the aphid tribe, and the number of replications performed in experiments for each parasitoid species are reported in Table 1.

**Table 1:** Aphid tribe, species, their color, host plants, and the number of replicates for *Aphelinus abdominalis*, *Aphidius ervi,* and *Diaeretiella rapae,* respectively

Tribe	Species	Aphid color	Host plant species	Replication		
				Preference	Performance	Endosymbiont presence
Aphidini	*Aphis fabae*	Black	Bean (*Vicia fabae*)	(32, 52, 30)	(n/a, 64, n/a)	(6)
	*Aphis gossipy*	Yellow	Squash (*Cucurbita moschata*)	(32, 47, 30)	(n/a, 69, 59)	(5)
	*Aphis craccivora*	Black	Bean (*Vicia fabae*)	(31, 70, 31)	(41, 45, n/a)	(5)
	*Aphis nerii*	Yellow	Milkweed (*Asclepias* sp.)	(37, 63, 31)	(58, 46, 70)	(5)
	*Rhopalosiphum padi*	Black	Wheat (*Hordeum vulgare*)	(33, 67, 32)	(66, 59, 73)	(4)
	*Schizaphis graminum*	Green	Wheat (*Hordeum vulgare*)	(32, 58, 40)	(63, 53, 68)	(5)
*Macrosiphini*	*Brevicoryne brassicae*	Green	Cabbage (*Brassica oleracea*)	(33, 109, 46)	(65, 56, 76)	(5)
	*Myzus persicae*	Green or Red	Cabbage (*Brassica oleracea*)	(66, 127, 80)	(181, 99, 171)	(10)
	*Sitobion avenae*	Green	Wheat (*Hordeum vulgare*)	(37, 59, 45)	(82, 54, 78)	(5)
	*Metopolophium dirhodum*	Yellow	Wheat (*Hordeum vulgare*)	(32, 55, 30)	(80, 52, n/a)	(6)
	*Macrosiphum euphorbiae*	Green	Potato (*Solanum tuberosum*) Tomato (*Solanum lycopersicum*)	(64, 107, 60)	(221, 138, 118)	(10)
	*Acyrthosiphon pisum*	Green	Bean (*Vicia fabae*)	(32, 41, 30)	(80, 42, 61)	(6)

Note

All aphid species tested belonged to the family Aphididae (subfamily Aphidinae), Blackman and Eastop (2006). All aphid colonies were initiated from ≥200 collected individuals naturally colonizing fields in France during 2013–2014 and infesting their respective host species. Two strains of *M. euphorbiae* were maintained on their respective collection plant species: *S. tuberosum* and *S. lycopersicum* (indicated as P and T for potato and tomato in the text, respectively). Two strains of *M. persicae* were used (one green and one red). All aphids were maintained (for 35–45 generations) on their host plant in a ventilated cage (60 × 60 × 60 cm) covered by mesh, under controlled conditions (23 ± 2°C, RH 65 ± 5% and photoperiod 16:8 hr L:D). n/a means that data are nonavailable due to the unsuccessful sting of the parasitoids in these aphid species.

*Aphelinus abdominalis* and *A. ervi* colonies were initiated from parasitoids naturally colonizing *Macrosiphum euphorbiae* on *S. lycopersicum* in the INRA Sophia Antipolis greenhouse complex (57 [21 males and 36 females] and 61 individuals [19 males and 42 females], respectively). *Diaeretiella rapae* colony was initiated from parasitoids naturally colonizing *Brevicoryne brassicae* in Brittany (29 [12 males and 17 females]). They were reared for 4–5 generations in the laboratory before starting the experiments. All parasitoids were maintained on their principal hosts (most frequent and/or suitable host): *Acyrthosiphon pisum* for *A. abdominalis* (Hullé, Turpeau, & Chaubet, 2006; Pons, Lumbierres, Antoni, & Stary, 2011) and *A. ervi* (Kavallieratos et al., 2004; Nguyen et al., 2018) wasps and *B. brassicae* for *D. rapae* (Desneux, Rabasse, Ballanger, & Kaiser, 2006; Kavallieratos et al., 2004) in climatic cabinets (23 ± 2°C, RH 65 ± 5% and photoperiod 16:8 L:D hr). Before experiments, the parasitized aphids were retrieved at the mummy stage and isolated in plastic Petri dishes. After adult emergence, females were mated and fed a honey solution (50% water + 50% honey) for at least 24 hr. Parasitoids used for all experiments were 24–48 hr old, used only once, and had never been in contact with plants or aphids (i.e., no experience before being tested).

### 2.2 Experiment 1: Parasitoid preference and performance measurements

Parasitoid preference. Preference for the different host species for all three parasitoid species was estimated by observing parasitoid behavior when they encountered individuals of the different aphid species tested. Three parasitoid behavioral steps were identified: detection, acceptance, and oviposition (see Desneux, Barta, Hoelmer, et al., 2009). For *A. ervi* and *D. rapae*, detection was defined as physical contact between aphid and parasitoid, followed by antennal palpation. Acceptance was described as the parasitoid abdomen bending underneath its thorax in the direction of the aphid, and sting was described as the introduction of the ovipositor into the aphid. In case of *A. abdominalis* wasps, effective detection was described as right‐left bounces behind the aphid followed by parasitoid acceptance, i.e., 180° rotation of the female, and the start of ovipositor use. A sting was described as an ovipositor introduction into aphids lasting at least 20 s and not ending as a result of aphid defensive behaviors (Wahab, 1985). Aphid defensive behaviors were also recorded, and three behaviors were considered defensive: kicking, cornicle secretion, and escape. For the analyses, all defensive behaviors were grouped.

For each replicate, one leaf of one host plant was placed upside down under a binocular magnifier (8×). One individual from one aphid species was placed on the leaf with a fine brush. After 5 min of establishment, one mated female parasitoid was introduced. When the parasitoid touched the leaf, the observation began and the parasitoid's behavior was noted over 5 min for *A. ervi* and *D. rapae* (short stinging time) and over 10 min for *A. abdominalis* (long stinting time, Wahab, 1985). The preference experiment was stopped after the 5 or 10 min or when the parasitoid exhibited oviposition behavior. Each parasitoid and aphid species was tested randomly every experimental day.

Aphid size is known to have an impact on the parasitoid host selection process (Wyckhuys et al., 2008). Hence, the aphids used in the experiment were all of the equivalent size of 3rd‐ and 2nd‐instar *A. pisum* for *A. abdominalis* and *A. ervi* wasps, respectively, and equivalent to 3rd‐instar *B. brassicae* for *D. rapae*, i.e., the known instar preferred by these parasitoids for oviposition (Henry, Gillespie, & Roitberg, 2005; Khakasa, Mohamed, Lagat, Khamis, & Tanga, 2016; Wahab, 1985).

Parasitoid performance. The physiological host range was established by monitoring parasitoid development in the different aphid species. Aphids stung in part 1 of the experiment were isolated in plastic Petri dishes (Ø 9 cm × H 1.7 cm) on one leaf of their respective host plant in a climatic room at 23 ± 1°C, RH 65 ± 5% and photoperiod of 16:8 hr L:D. In order to increase the sample size for the performance analyses, additional replicates were performed under the same conditions as in part 1 without recording parasitoid behavior.

Parasitoid development within the host was monitored at four different times. The aphids were (a) dissected within 1 hr after being stung to check the presence of parasitoid eggs, under a binocular microscope at 100× magnification (to adjust the sting rate), (b) dissected after 4 days to measure survival of immature parasitoids under a binocular microscope at 40× magnification, and (c) checked at 7 days to monitor aphid mummification (number of replicate Table 1). The emergence rate of mummies and the sex ratio of emerged adults were recorded as well.

### 2.3 Experiment 2: Presence of secondary endosymbionts in aphid

To evaluate the impact of aphid secondary endosymbionts on the development of juvenile parasitoids, each aphid colony was screened using PCR (Materials and Methods S1) to detect the presence of nine facultative symbiont genera (Table S1) that are known to interact with aphids (Desneux et al., 2018; Ferrari & Vavre, 2011): *Arsenophonus* sp.*, Hamiltonella defensa* (T‐type), *PAXS* (Pea‐aphid X‐type symbiont)*, Regiella insecticola* (U‐type), *Rickettsia* sp.*, Rickettsiella* sp.*, Serratia symbiotica* (R‐type), *Spiroplasma* sp.*, Wolbachia* sp (number of replicates for each aphid colony detailed Table 1). *Hamiltonella defensa* was found in all six *A. fabae* individuals tested and in 4 of the 6 *Metopolophium dirhodum* individuals tested. *Regiella insecticola* was found in all six *M. dirhodum* individuals tested. *Arsenophonus* sp., PAXS, *Rickettsia* sp., *Rickettsiella* sp., *Serratia symbiotica*, *Spiroplasma* sp.*,* and *Wolbachia* sp. were not found in any of the aphid species screened.

### 2.4 Data analysis

All statistical analyses were carried out using R version 3.2.2 (R Core Team 2017). The parasitoid behavior was analyzed using generalized linear models (GLMs) based on a binomial distribution. They were used (a) to compare parasitoid behaviors, i.e., detection, acceptance, and sting rates among aphid species, (b) to analyze the effect of aphid defensive behaviors on sting rate, and (c) to analyze the effect of aphid color, host plant, and aphid tribe on the proportion of aphids stung by the various parasitoids. The parasitoid performance was also analyzed using GLMs based on a binomial distribution. They were used (a) to compare proportion of egg, larvae, mummy, and adult parasitoids recorded among all aphid species; (b) to compare parasitoid mortality across the different development stages in each aphid species, that is, egg, larvae, mummy, and adult stages; and (c) to analyze the effect of aphid color, host plant, and aphid tribe on the proportion of emerged parasitoids. When required, the GLMs were followed by a multicomparison test (Tukey, package “multcomp”). The deviation from a 0.5 sex ratio for only the parasitoids that produced ≥ 10 offspring (number enable to reasonably estimate a sex ratio) was tested with permuted Fisher's exact test (with the Bonferroni adjustment method). Finally, to analyze the relationship between the preference (sting rate) and the performance (emergence rate) of parasitoids, a comparative analysis of independent contrasts (calculation of phylogenetically independent variables, as described by Felsenstein (1985)) was used (CAIC, package “caper”).

In addition to testing for occurrence of a PPH relationship, we also calculated the index of host specificity $S_{\text{TD}}^{\text{*}}$ from Poulin and Mouillot (2005) to better characterize host specificity of tested parasitoids. Contrary to the former S_TD_ from Poulin and Mouillot (2003), the $S_{\text{TD}}^{\text{*}}$ is a value which depends on (a) the prevalence of the parasitoid on the various hosts and (b) the position of these host species within a taxonomic hierarchy (Poulin & Mouillot, 2005). The smaller the value, the more specialist the parasitoid. The $S_{\text{TD}}^{\text{*}}$ was computed using the program TaxoBiodiv2 (Poulin & Mouillot, 2005), which considers a taxonomic tree of hosts built based on family, tribe, genus, and species (Blackman & Eastop, 2006) and the proportions of parasitoid adult emergence for each species in every aphid species. It was done for the generalist parasitoids *A. abdominalis*, *A. ervi*, and *D. rapae*, as well as for three other parasitoid species previously analyzed in the laboratory: *Binodoxys communis* (Desneux, Barta, Hoelmer, et al., 2009) and *Binodoxys koreanus* (Desneux, Starý, et al., 2009) described as specialist aphid parasitoids as well as *Lysiphlebus testaceipes* described as moderate specialist aphid parasitoids (from France and from United States; Monticelli, 2018).

## 3 RESULTS

For each parasitoid species tested, we tested the impact of the aphid colony, the host plant, the endosymbiont presence, the aphid color, and the aphid tribe on both the parasitoid preference and performance (Table 1).

### 3.1 Parasitoid preference

For *Aphelinus abdominalis*, the proportion of aphid individuals detected by the parasitoid for each aphid species ranged from 0.91 to 1.00 and was not significantly different (Tables 2 and 3). Three aphid species, *A. craccivora*, *A. fabae* and *A. gossypii*, were significantly less accepted and stung by the parasitoid than *A. pisum, A. nerii*, *M. euphorbiae* (P and T), and *Sitobion avenae*. *Aphelinus abdominalis* stung less black than green aphids and stung mainly aphids from the Macrosiphini tribe. The presence of secondary endosymbiont or the different host plants tested did not impact their preference (Tables 3 and S2). Due to the very low number of *A. craccivora, A. fabae,* and *A. gossypii* stung by *A. abdominalis,* these aphid species could not be considered in the subsequent performance assessment.

**Table 2:** Proportion of aphids detected, accepted, and stung by *Aphelinus abdominalis* (Aa), *Aphidius ervi* (Ae), and *Diaeretiella rapae* (Dr), respectively, upon the encounter of different aphid species

Aphid species/Parasitoid species	Proportion of aphids detected^a^			Proportion of aphids accepted			Proportion of aphids stung		
	Aa^a^	Ae	Dr	Aa	Ae	Dr	Aa	Ae	Dr
*Acyrthosiphon pisum*	*1.00*	***1.00 a***	**1.00 a**	***0.97* a**	***0.95* a**	**0.83 ae**	***0.85* a**	***0.89* ab**	**0.65 ab**
*Aphis craccivora*	1.00	**1.00 a**	0.58 c	0.48 b	0.5 b	0.19 cd	0.05 c	0.30 ce	0.13 c
*Aphis fabae*	0.94	0.92 b	0.83 bc	0.34 b	0.48 b	0.3 bcd	0.31 bc	0.29 ce	0.17 c
*Aphis gossypii*	0.91	0.96 b	0.93 b	0.34 b	**0.77 ab**	0.47 bde	0.25 bc	**0.55 abcd**	0.24 bc
*Aphis nerii*	1.00	**0.98 ab**	**0.97 a**	**0.89 a**	**0.68 ab**	**0.74 ae**	**0.75 a**	**0.47 abcd**	0.34 bc
*Brevicoryne brassicae*	0.97	0.96 b	***1.00 a***	**0.73 ab**	0.57 b	***0.98* a**	**0.55 ab**	0.10 e	***0.87* a**
*Macrosiphum euphorbiae (P)*	0.97	**1.00 a**	0.77 bc	**0.94 a**	**0.96 a**	0.43 bcde	**0.88 a**	**0.86 a**	0.14 c
*Macrosiphum euphorbiae (T)*	0.97	**1.00 a**	**0.93 a**	**1.00 a**	**0.88 a**	0.37 bcd	**0.88 a**	0.48 c	0.17 c
*Metopolophium dirhodum*	0.97	**0.96 ab**	0.77 bc	**0.81 a**	**0.93 a**	0.23 bc	**0.69 ab**	0.56 bc	0.13 c
*Myzus persicae (Green)*	0.97	**0.97 ab**	**1.00 a**	**0.84 a**	**0.94 a**	**0.79 ae**	**0.64 ab**	**0.71 ad**	**0.64 ab**
*Myzus persicae (Red)*	0.97	**1.00 a**	0.93 b	**0.69 ab**	**0.93 a**	**0.63 ab**	**0.62 ab**	**0.55 abcd**	0.39 bc
*Rhopalosiphum padi*	1.00	0.92 b	0.94 b	**0.82 a**	0.55 b	0.47 bcde	**0.59 ab**	0.28 ce	0.30 bc
*Schizaphis graminum*	0.97	0.91 b	**0.98 ab**	**0.81 a**	**0.71 ab**	**0.95 a**	**0.63 ab**	0.27 ce	**0.76 a**
*Sitobion avenae*	1.00	**1.00 a**	**1.00 a**	**0.89 a**	**0.92 a**	**0.84 a**	**0.78 a**	**0.67 ad**	**0.54 abc**

Note

For each parasitoid species, proportions followed by the same letter are not significantly different (GLMs followed by a multicomparison test) and the proportions of the rearing host (*A. pisum* for *A. abdominalis* and *A. ervi* and *B. brassicae* for *D. rapae*) are indicated in italics. The most detected, accepted, and stung aphid species are indicated in bold text.

a No significant difference in the proportion of aphids detected by *A. abdominalis* among the species.

**Table 3:** Effect of the aphid colony, the host plant species, the presence of endosymbiont, the aphid color, and the aphid tribe on the parasitoid preference (proportion of aphids detected, accepted, and oviposited by the parasitoids) and the parasitoid performance (proportion of parasitoid larvae, of mummified aphid, and adult emerged). Significant effects of the various factors on the parasitoid preference and performance traits are indicated in bold text

	Parasitoid parameters (%)	Aphid colony				Plant species				Endosymbiont presence				Aphid color				Aphid tribe			
		*χ* ^2^	*df*	*p* value	Dp	*χ* ^2^	*df*	*p* value	Dp	*χ* ^2^	*df*	*p* value	Dp	*χ* ^2^	*df*	*p* value	Dp	*χ* ^2^	*df*	*p* value	Dp
*Aphelinus abdominalis*	Detection	15.5	13	0.276	1.0	8.1	6	0.170	0.9	1.4	1	0.279	1.2	1.4	3	0.744	1.1	0.6	1	0.423	1.0
	Acceptance	112.5	13	**<0.001**	1.0	71.9	6	**0.022**	4.8	13.4	1	0.201	8.2	46.8	3	0.056	6.2	34.1	1	**0.017**	6.0
	Oviposition	111.3	13	**<0.001**	1.0	60.5	6	**0.029**	4.3	3.8	1	0.501	8.5	48.4	3	0.045	6.0	39.6	1	**0.007**	5.5
	Larvae	88.6	10	**<0.001**	1.0	73.3	5	**<0.001**	3.3	1.9	1	0.675	10.6	26.8	3	0.333	7.9	30.4	1	**0.022**	5.8
	Mummy	141.7	10	**<0.001**	1.0	87.8	5	0.132	10.4	3.2	1	0.633	14.2	9.5	3	0.913	18.0	40.5	1	0.063	11.7
	Adult	123.9	10	**<0.001**	1.0	65.0	5	0.272	10.2	6.7	1	0.441	11.3	4.5	3	0.959	14.8	27.2	1	0.098	10.0
*Aphidius ervi*	Detection	31.8	13	**0.003**	1.0	10.4	6	0.627	2.4	2.6	1	0.279	2.2	5.8	3	0.438	2.1	7.2	1	0.030	1.5
	Acceptance	155.9	13	**<0.001**	1.0	39.9	6	0.850	15.0	0.9	1	0.781	11.9	73.1	3	**0.031**	8.2	64.1	1	**0.007**	8.7
	Oviposition	196.9	13	**<0.001**	1.0	39.9	6	0.928	20.9	0.5	1	0.854	14.9	69.4	3	**0.020**	7.1	55.4	1	**0.008**	7.8
	Larvae	23.3	12	**0.025**	1.0	4.6	6	0.954	2.9	3.2	1	0.170	1.7	1.1	3	0.927	2.3	1.6	1	0.347	1.8
	Mummy	96.1	12	**<0.001**	1.0	10.9	6	0.992	13.8	24.4	1	**0.039**	5.7	60.6	3	**<0.001**	3.2	49.6	1	**<0.001**	3.8
	Adult	87.5	12	**<0.001**	1.0	14.8	6	0.974	11.9	19.3	1	0.071	5.9	51.5	3	**0.002**	3.4	38.7	1	**0.001**	3.7
*Diaeretiella rapae*	Detection	72.1	13	**<0.001**	1.0	27.7	6	0.578	5.8	8.4	1	0.267	6.8	24.4	3	0.239	5.8	4.8	1	0.373	6.0
	Acceptance	157.7	13	**<0.001**	1.0	51.8	6	0.714	13.9	35.6	1	0.050	9.3	68.5	3	0.054	9.0	8.6	1	0.381	11.2
	Oviposition	129.9	13	**<0.001**	1.0	58.5	6	0.433	9.9	17.6	1	0.154	8.6	52.8	3	0.061	7.2	9.1	1	0.328	9.5
	Larvae	173.6	10	**<0.001**	1.0	173.5	6	**<0.001**	0.0	NA				27.5	3	0.640	16.4	21.8	1	0.199	13.2
	Mummy	152.2	10	**<0.001**	1.0	125.0	6	**<0.001**	5.1	NA				23.5	3	0.661	14.8	5.4	1	0.515	12.8
	Adult	127.5	10	**<0.001**	1.0	103.9	6	**<0.001**	4.3	NA				19.2	3	0.661	12.0	11.3	1	0.279	9.6

Abbreviation: Dp, dispersion parameter.

For *Aphidius ervi*, the proportion of detected individuals of each aphid species ranged from 0.91 to 1.00 and varied significantly among the aphid species (Table 2 and 3). Five aphid species, *A. craccivora, A. fabae, B. brassicae, R. padi,* and *S. graminum,* were significantly less accepted and stung than *A. pisum, M. euphorbiae* (P), *M. persicae* (green strain), and *S. avenae*. *Aphidius ervi* accepted and stung less black than green aphids and aphids from the Aphidini tribe, regardless of the host plant species tested (Tables 3 and S2). *Brevicoryne brassicae* was stung by *A. ervi* at the lowest level and it could not be considered in the followed performance assessment.

For *D. rapae*, the proportion of aphid individuals detected ranged from 0.58 to 1.00 and varied significantly among the aphid species (Tables 2 and 3). Three aphid species (*A. craccivora, A. fabae,* and *M. dirhodum*) were significantly less accepted and stung than *A. pisum*, *B. brassicae, M. persicae* (green strain), and *S. graminum*. *Macrosiphum euphorbiae (*P and T*)* were not less accepted that these aphid species but were less stung. *Diaeretiella rapae* preference was similar regardless of aphid color, tribe, or host plant (Tables 3 and S2). Due to the very low number of *A. craccivora*, *A. fabae,* and *M. dirhodum* stung by *D. rapae,* these aphid species could not be considered in the subsequent performance assessment.

**Figure 1:**
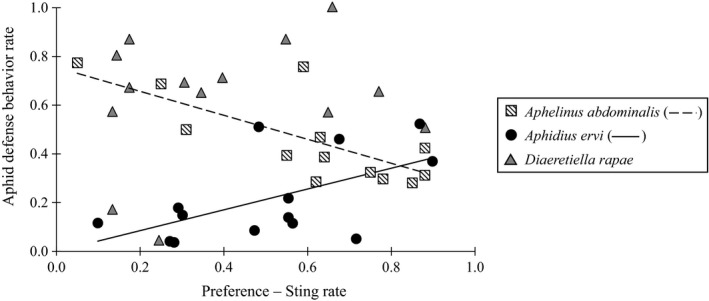
Relationship between the proportion of aphids stung in each aphid species by every parasitoid species tested (*Aphelinus abdominalis, Aphidius ervi,* and *Diaeretiella rapae*) and the proportion of aphid defensive behaviors exhibited by the aphid when it encountered these parasitoid species. The dashed line represents the negative relationship in *A. abdominalis* ($\chi_{1}^{2}\;$ = 53.1, *p* < 0.001), and the continuous line represents the positive relationship in *A. ervi* ($\chi_{1}^{2}\;$ = 53.1, *p* < 0.001). No relationship was observed in *D. rapae* ($\chi_{1}^{2}\;$ = 3.3, *p* > 0.07)

When attacked by *A. abdominalis*, *A. ervi,* and *D. rapae*, respectively 45%, 20%, and 62% of aphids exhibited defensive behaviors. *Acyrthosiphon pisum, M. euphorbiae,* and *S. avenae* exhibited significantly higher defensive behaviors (57% rate of defensive reaction) than *A. craccivora, A. gossypii, A. nerii, M. dirhodum, M. persicae* (green strain), and *S. graminum* (34% rate of defensive reaction). There was a negative relationship between the proportion of aphids stung and exhibiting defensive behaviors when attacked by *A. abdominalis* ($\chi_{1}^{2}\;$ = 53.1, *p* < 0.001) (Figure 1). By contrast, this relationship was positive for *A. ervi* wasps ($\chi_{1}^{2}\;$ = 53.1, *p* < 0.001) and no relationship was observed for *D. rapae* ($\chi_{1}^{2}\;$ = 3.3, *p* > 0.07). The occurrence of aphid defensive behaviors thus varied depending on the parasitoid species ($\chi_{1}^{2}\;$ = 270.9, *p* < 0.001) and the aphid species ($\chi_{13}^{2}\;$ = 99.3, *p* < 0.001).

### 3.2 Parasitoid performance

**Figure 2:**
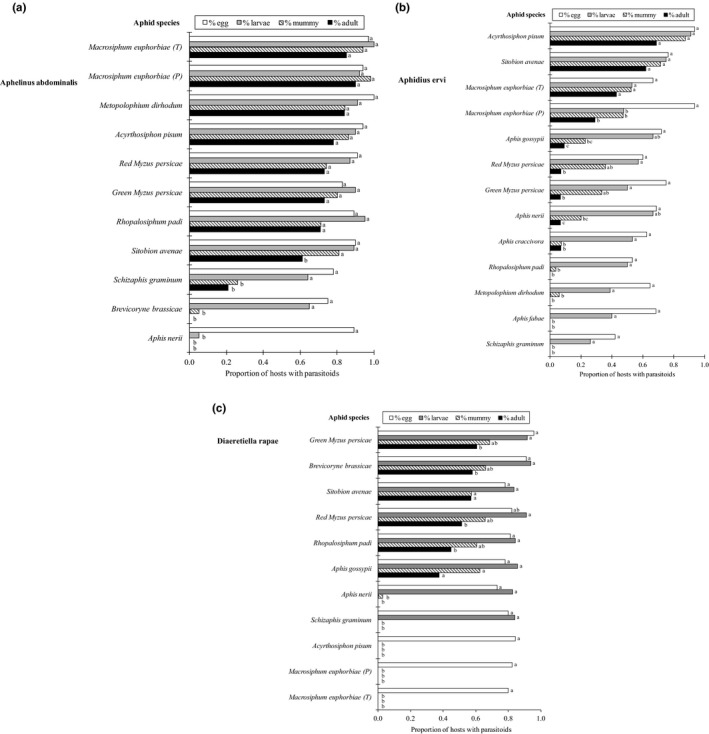
Proportion of stinging aphids that contained an egg (dissection after stung), contained a larva (dissection after 4 days), mummified (after 10 days), and produced an adult parasitoid for (a) *Aphelinus abdominalis,* (b) *Aphidius ervi,* and (c) *Diaeretiella rapae* (experiment 2). For each aphid species, bars followed by the same letter are not significantly different (generalized linear models followed by multicomparison test)

For *Aphelinus abdominalis*, the offspring emergence rate ranged from 0.71 to 0.90, the highest being in *A. pisum, M. euphorbiae* (on potato and tomato plants), *M. dirhodum*, *M. persicae* (green and red strains), *R. padi,* and *S. avenae* (Table 3, Figure 2a). The other aphid species were grouped based on the stage at which the parasitoid development failed. First, in *Aphis nerii*, mortality was significantly higher between egg and larval stages ($\chi_{3}^{2}\;$ = 63.0, *p* < 0.001). Second, in *B. brassicae* and *S. graminum*, significant mortality was observed between larval and pupal stages ($\chi_{3}^{2}\;$ = 48.7 and 19.7, respectively, all *p* < 0.001). Finally, in *S. avenae,* mortality was observed during the pupal stage ($\chi_{3}^{2}\;$ = 9.7, *p* = 0.022). *Aphelinus abdominalis* performance did not vary depending on the aphid colors and/or host plants (Tables 1, 3 and S2). However, a higher number of larvae survived in aphid species belonging to the Macrosiphini tribe (Table 3). The sex ratio was male‐biased in *M. dirhodum*, *M. persicae* (green and red strains), and *R. padi* (all *p* < 0.001) and female‐biased in *M. euphorbiae* on tomato (*p* = 0.036; Table 4).

**Table 4:** Female sex ratio (proportions: females/adult emerged) for each parasitoid species developing on different hosts.

Aphid species	Female sex ratio (proportion females)		
	*Aphelinus abdominalis*	*Aphidius ervi*	*Diaeretiella rapae*
*Acyrthosiphon pisum*	0.67	0.73	n/a
*Aphis gossypii*	n/a	n/a	n/a
*Brevicoryne brassicae*	n/a	n/a	0.59
*Macrosiphum euphorbiae (P)*	0.44	0.60	n/a
*Macrosiphum euphorbiae (T)*	0.76*	0.56	n/a
*Metopolophium dirhodum*	0.04***	n/a	n/a
*Myzus persicae (red)*	0***	n/a	0.50
*Myzus persicae (green)*	0.09***	n/a	0.61
*Rhopalosiphum padi*	0.05***	n/a	0.59
*Sitobion avenae*	0.32	0.54	0.55
*Aphis fabae*	n/a	n/a	n/a
*Aphis craccivora*	n/a	n/a	n/a
*Schizaphis graminum*	n/a	n/a	n/a
*Aphis nerii*	n/a	n/a	n/a

Note

n/a means that data are nonavailable due to the unsuccessful development of the parasitoids in these aphid species.

* *p* < 0.05,

*** *p* < 0.001 (deviation from a 0.5 sex ratio).

For *Aphidius ervi,* the highest adult emergence rate was 0.62 and 0.69, respectively, for *S. avenae* and *A. pisum* (Table 3, Figure 2b). The other aphid species could be grouped upon the stage at which the parasitoid development failed. First, in *M. euphorbiae on* potato, mortality was significant higher between the egg and larval stages ($\chi_{3}^{2}\;$ = 24.7, *p* < 0.001). Second, in *A. craccivora*, *A. fabae*, *A. gossypii*, *A. nerii*, *M. dirhodum, R. padi,* and *S. graminum,* significant mortality was observed between the larval and pupal stages ($\chi_{3}^{2}\;$ > 15.0, respectively, all *p* < 0.001). Finally, in *M. persicae* (green and red strains), mortality was primarily observed during the pupal stage ($\chi_{3}^{2}\;$ = 17.8, *p* < 0.001). *Aphidius ervi* produced a higher proportion of offspring in the green aphids compared to the black ones, in the aphids belonging to the Macrosiphini tribe and in the aphids that did not harbor a secondary endosymbiont (Tables 1 and 3). The host plant did not modulate the performance of *A. ervi* (Tables 1, 3, and S2). The *A. ervi* sex ratio was similar to 50:50 in all aphid species tested (all *p* > 0.05; Table 4).

For *D. rapae,* the adult emergence rate ranged from 0.38 to 0.61 and the highest was in *A. gossypii, B. brassicae, M. persicae* (green and red strains), *R. padi,* and *S. avenae* (Table 3, Figure 2c). The other aphid species could be grouped upon the stage at which parasitoid development failed. First, mortality was significant between the egg and larval stages in *A. pisum* and *M. euphorbiae* ($\chi_{3}^{2}\;$ = 63.9, 58.8, all *p* < 0.001). Second, significant mortality was observed between the larval and pupal stages in *A. nerii* and *S. graminum* ($\chi_{3}^{2}\;$ = 83.9 and 72.0, respectively, all *p* < 0.001). Finally, mortality was observed during the pupal stage in *B. brassicae*, *M. persicae* (green strain), and *R. padi* ($\chi_{3}^{2}\;$ = 13.9, 16.3, and 11.9, *p* = 0.003, 0.0009, and 0.007, respectively). *Diaeretiella rapae* performance was modulated by the host plant, and a lower offspring proportion was observed in aphid species maintained on *Asclepias*, bean, potato, and tomato than on cabbage, squash, and wheat (Tables 1, 3, and S2). However, the aphid color and tribe did not modulate the parasitoid performance (Tables 1 and 3). The *D. rapae* sex ratio was similar to 50:50 in all aphid species (all *p* > 0.05; Table 3).

### 3.3 The PPH relationship

**Figure 3:**
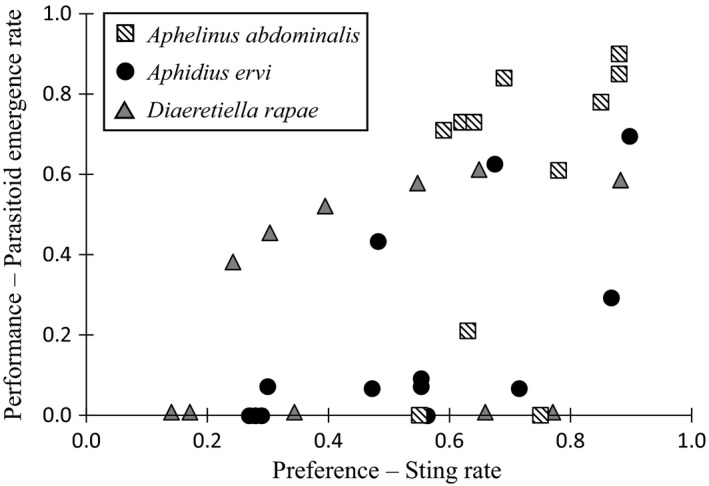
Relationship between the sting rate (preference) and the emergence rate (performance) when three generalist parasitoids (*Aphelinus abdominalis, Aphidius ervi,* and *Diaeretiella rapae*) encountered twelve aphid species. Relationship between the proportion of aphids stung in each aphid species by every parasitoid species tested (*A. abdominalis, A. ervi,* and *D. rapae*) and the proportion of emerged adults of the aphids stung. No relationship was observed in *A. abdominalis* (*F*_1,7_ = 0.99, *p* = 0.353, *R* ^2^ = 0.12), *A. ervi* (*F*_1,9_ = 3.96, *p* = 0.078, *R* ^2^ = 0.31), and *D. rapae* (*F*_1,7_ = 0.21, *p* = 0.663, *R* ^2^ = 0.03)

The preference–performance hypothesis (PPH) have been found in specialist arthropod, but it has been argued that such relationships may not be common in generalist ones. In this study, no significant relationship was found between the preference (sting rate) and the performance (emergence rate) of *A. abdominalis* (*F*_1,7_ = 0.99, *p* = 0.353, *R* ^2^ = 0.12), *A. ervi* (*F*_1,9_ = 3.96, *p* = 0.078, *R* ^2^ = 0.31), and *D. rapae* (*F*_1,7_ = 0.21, *p* = 0.663, *R* ^2^ = 0.03; Figures 3 and S1).

### 3.4 Index of host specificity

**Figure 4:**
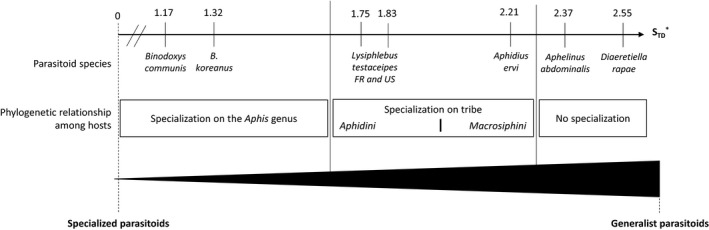
Host specificity index ($S_{\text{TD}}^{\text{*}}$) values and ranking of the parasitoids according to their degree of specialization

*Aphelinus abdominalis* and *D. rapae* are the more generalist aphid parasitoid considered in this study and their $S_{\text{TD}}^{\text{*}}$ are 2.37 and 2.55, respectively (Figure 4). *Aphidius ervi* and *L. testaceipes* (from previous study) are oligophagous species and their $S_{\text{TD}}^{\text{*}}$ are 2.21, 1.75, and 1.83, respectively. Finally, *B. communis* and *B. koreanus* (from previous study) were the most specialized species considered in our study and their $S_{\text{TD}}^{\text{*}}$ are, respectively, 1.17 and 1.32.

## 4 DISCUSSION

Generalist parasitoids are known to use a broad set of cues to identify potential hosts, and this may alter the relationship between their preference and performance traits (Mackauer et al., 1996; Vet & Dicke, 1992). Testing such a PPH relationship in three generalist aphid parasitoids on twelve aphid host species revealed that such a relationship is absent in *A. abdominalis, A. ervi,* and *D. rapae*. All three parasitoids showed low behavioral selectivity when encountering potential host species (preference traits), and thus, their host range was primarily dictated by the actual host suitability for their offspring development. Host suitability in two particular aphid species was likely affected by the presence of two detected secondary endosymbionts (von Burg, Ferrari, Muller, & Vorburger, 2008; Oliver et al., 2003). In parallel, the calculated $S_{\text{TD}}^{\text{*}}$ values were consistent with the results from PPH assessments (present study and our previous ones) and enabled the categorization of the aphid parasitoids as generalist (*D. rapae* and *A. abdominalis*), oligophagous (*A. ervi* and *L. testaceipes*), or more specialized (*B. communis* and *B. koreanus*) species.

### 4.1 Preference traits

As expected for generalist parasitoids, the three species tested showed low host selectivity, i.e., they stung all aphid species encountered (at least in some extent) regardless to the host plant species or the endosymbiont presence. However, *A. abdominalis* and *A. ervi* might show a preference for the green aphids over the black ones (though confounding effect could not be excluded, for example, all Macrosiphini aphids tested were greenish, whereas all the black aphids belonged to the Aphidini tribe). Still, visual cues are used by many organisms to evaluate and select resources (Bell, 1991), and green aphids are known to be well detected by aphid parasitoids; *Aphidius rhopalosiphi*, *Monoctonus paulensis*, and *Praon pequodorum* preferred green aphids (Michaud & Mackauer, 1994, 1995). In addition, *A. abdominalis* and *A. ervi* attacked significantly more aphids from the Macrosiphini tribe. Overall, they stung all the aphid species from the Macrosiphini tribe encountered at high proportions and some species from the Aphidini tribe, such as *R. padi* and *S. graminum*. Closely related species may share characteristics recognized by parasitoid to select their hosts (Bell, 1991; Harvey et Pagel, 1991; Ives & Godfray, 2006; Michaud & Mackauer, 1994). In our study, we did not consider the beginning of the host selection process which may occur at long distance, i.e., habitat location (according to the definition by Vinson, 1985) and doing so we could have missed a part of the behavioral selectivity. However, the host selection process by generalist parasitoids mostly relies on semiochemicals originating from the hosts themselves (Becker et al., 2015) and notably in aphid parasitoids (Hatano, Kunert, Michaud, & Weisser, 2008).

Aphid defensive behaviors are known to potentially affect oviposition behavior of various aphid parasitoids and to reduce the parasitoid host range (Desneux, Barta, Hoelmer, et al., 2009; Kouamé & Mackauer, 1991; Wyckhuys et al., 2008). To avoid aphid defensive behaviors, some parasitoids, including *A. ervi* and *D. rapae,* exhibited a “quick” sting syndrome (Desneux, Barta, Delebecque, & Heimpel, 2009; Völkl & Mackauer, 2000). In this study, *D. rapae* did not show a relationship between its sting rate and the aphid defense rate, suggesting that *D. rapae* is able to avoid aphid defensive behaviors. A positive relationship was found between the *A. ervi* sting rate and the aphid defenses rate induced by this parasitoid species; aphid defenses such as cornicle secretions and defensive movements might be used by *A. ervi* as chemical and physical cues, respectively (Battaglia et al., 2000). Contrarily, there was a negative relationship between the proportion of aphid species stung by *A. abdominalis* and the proportion of aphid defensive behaviors. *Aphelinus abdominalis* demonstrated a sting time ranging between 20 and 60 s, about four times that of the two other parasitoid species tested. This suggests that aphid defensive behaviors may disturb females during their stinging event (De Farias & Hopper, 1999; Wahab, 1985). Secondly, success of aphid defenses depends on the relative size of the attacking parasitoid versus the aphid. *Aphelinus abdominalis* is two times smaller than *A. ervi* and *D. rapae,* whose size allows them to attack aphids more easily (Le Ralec et al., 2010).

### 4.2 Performance traits

*Aphelinus abdominalis* and *D. rapae* were able to produce offspring with high prevalence in aphids from both Aphidini and Macrosiphini tribes, whereas *A. ervi* was able to produce offspring in aphid species mainly from the Macrosiphini. As previously, closely related species can share characteristics used by parasitoids to complete their development in their hosts (Harvey et Pagel, 1991; Ives et Godfray, 2006), suggesting that *A. ervi* is specialized on aphids belonging to the Macrosiphini tribe (as in Zepeda‐Paulo, Ortiz‐Martínez, Figueroa, & Lavandero, 2013).

Different physiological and ecological factors could then provide aphids with resistance against immature parasitoids and could modify parasitoid host range (Monticelli, 2018). The main sources of resistance include poor parasitoid ability to control host metabolism (Godfray, 1994), the aphid host plant (Desneux, Barta, Hoelmer, et al., 2009), the presence of secondary endosymbionts (Oliver, Moran, & Hunter, 2005; Vorburger, Gehrer, & Rodriguez, 2009), the aphids' ability to sequester toxic compounds (Desneux, Barta, Hoelmer, et al., 2009; Francis, Lognay, Wathelet, & Haubruge, 2001), and/or the host quality itself (Godfray, 1994; Kouamé & Mackauer, 1991).

The facultative endosymbionts present in aphids may compromise the successful development of parasitoids explaining the parasitoid mortality between the egg and larvae stage (Ferrari, Darby, Daniell, Godfray, & Douglas, 2004; McLean & Godfray, 2015; Oliver et al., 2005). Specifically, *Hamiltonella defensa* and *Regiella insecticola* (detected in *A. fabae* and *M. dirhodum* as reported by Henry, Maiden, Ferrari, & Godfray, 2015) associated with a toxin‐encoding bacteriophage (ASPE, Oliver, Degnan, Hunter, & Moran, 2009) are known to provide aphids protection against different natural enemies, such as parasitoids (Oliver et al., 2003). In this study, *A. abdominalis* was not impacted by the presence of secondary endosymbionts since it had 84% successful parasitism in *M. dirhodum* (as in McLean, Hrček, Parker, & Godfray, 2017 and Hopper et al., 2018). The presence of *R. insecticola* may, in the case of *Aphelinus* sp., induce a higher parasitism rate (Luo et al., 2017) or a higher fitness, for example, *Aphelinus glycinis* produced more and larger female adult progeny on infected than on uninfected aphids (Hopper et al., 2018). By contrast, a strong parasitoid larval mortality of *Aphidius ervi* was observed when it encountered *A. fabae* and *M. dirhodum*, suggesting that *H. defensa* and/or *R. insecticola* had a strong negative impact on *A. ervi* performance (as in Oliver et al., 2003, Vorburger et al., 2009). In addition, *A. ervi* is well known to parasite Macrosiphininae species such as *M. dirhodum* (Starý 1993, Kavallieratos et al., 2004), suggesting that the presence of *H. defensa* and/or *R. insecticola* reduces the *A. ervi* host range. Finally, *D. rapae* stung *A. fabae* and *M. dirhodum* at a low rate that does not permit an evaluation of the impact of *H. defensa* and *R. insecticola* on parasitoid performance. The impact of endosymbionts is variable depending on the aphid–parasitoid system considered and more studies are needed. Several studies have shown that endosymbionts confer protection only against the more specialized natural enemies and less against generalist ones (Asplen et al., 2014; Hrcek, McLean, & Godfray, 2016; Kraft, Kopco, Harmon, & Oliver, 2017; Parker, Spragg, Altincicek, & Gerardo, 2013), which support the hypothesis whereby *A. ervi* is more specialized than *A. abdominalis* and *D. rapae*.

The host plant may also contribute to a reduction in aphid parasitoid host range. *Aphelinus abdominalis* and *A. ervi* can parasitize aphids on multiple host plants, whereas *D. rapae* fails to develop in aphid species maintained on milkweed, bean, potato, and tomato. *Diaeretiella rapae* is an aphid parasitoid generalist and a habitat specialist (notably on *Brassicae* and *Gramineae,* Kavallieratos et al., 2004). In our study, *A. craccivora and A. fabae* were not stung by *D. rapae,* whereas when these aphid species are found on Brassicaceae, they are considered as suitable hosts for *D. rapae* (Alikhani, Rezwani, Starý, Kavallieratos, & Rakhshani, 2013; Kavallieratos et al., 2004), suggesting that bean modulates parasitoid performance.

The specialist aphid species *A. nerii* and *B. brassicae* are able to sequester cardenolide (Asclepias) and glucosinolate (cabbage), respectively (Desneux, Barta, Hoelmer, et al., 2009; Jones, Bridges, Bones, Cole, & Rossiter, 2001), and these toxic allelochemical molecules have a drastic impact on immature parasitoid survival (Desneux, Barta, Hoelmer, et al., 2009; Kos et al., 2012; Mooney, Jones, & Agrawal, 2008; Pratt, Pope, Powell, & Rossiter, 2008) and may explain the parasitoid mortality between the larvae and pupae stage. The three generalist parasitoids cannot successfully parasitize *A. nerii* aphids living on *Asclepias,* despite a high sting rate (up to 0.75) equal to that of other, nontoxic host plants. All developing parasitoids reached the larval stage and then died. *Diaeretiella rapae* is able to use glucosinolates (and/or related compounds) as long‐ and short‐distance kairomones for selecting its hosts (Bradburne & Mithen, 2000) as well as to promote effective development of its offspring in aphids such as *B. brassicae* (Kos et al., 2012). However, *A. abdominalis* cannot successfully parasitize *B. brassicae* despite a non‐negligible sting rate (0.55). *Myzus persicae* also feeds on cabbage but is not a glucosinolate‐sequestering aphid and excretes the glucosinolates in its honeydew, reducing the impact on parasitoid offspring development (Francis et al., 2001; Weber 1986). *Aphelinus abdominalis* exhibits successful development in *M. persicae* (0.73 adults emerged), suggesting that *A. abdominalis* is strongly affected by glucosinolates. Conversely, *A. ervi* did not sting *B. brassicae* and cannot complete its development in *M. persicae,* which is generally found to be a suitable host (Colinet, Salin, Boivin, & Hance, 2005; Kavallieratos et al., 2004), suggesting that aphid genotype could be involve in this failure to parasitize (Bilodeau, Simon, Guay, Turgeon, & Cloutier, 2013; von Burg et al., 2008). Aphid ability to sequester the toxic compounds from their host plant involves a high specialization of aphid species (Mooney et al., 2008) and only a few (up to four) parasitoid species can parasite *A. nerii* and *B. brassicae* (Kavallieratos et al., 2004), suggesting that strong circumventing mechanisms are needed for a parasitoid to adapt to aphid defense. Furthermore, sequestering is a general aphid defense against parasitoids as well as natural enemies (Omkar & Mishra, 2005; Toft & Wise, 1999).

Finally, host quality could also contribute to a high mortality of later parasitoid larval stages prior to emergence, or at least a modulation of their sex ratio (male‐biased; Godfray, 1994; Kouamé & Mackauer, 1991; Mackauer, 1986). Host species and age are the two most important factors determining parasitoid development. However, generalist parasitoids are less demanding in terms of host choice, as shown in the behavioral results of this study. For example, *S. graminum* caused a high larval mortality of the three parasitoid species (consistent with Desneux, Barta, Hoelmer, et al., 2009) and a high proportion of unemerged parasitoids of *A. ervi* were observed in *R. padi* despite high sting rates, indicating that *S. graminum* and *R. padi* are poor hosts for these parasitoid species. Furthermore, some parasitoids tend to place male eggs in unfavorable hosts (Godfray, 1994; Kochetova, 1978). In *A. abdominalis*, a male‐biased sex ratio was observed in *M. dirhodum*, *M. persicae* (green and red strains), *R. padi,* and *S. graminum*, suggesting that these aphid species are considered as low quality hosts for *A. abdominalis*.

### 4.3 The preference–performance relationship in generalist parasitoids

The meta‐analysis of Gripenberg et al. (2010) of the PPH relationship in phytophagous insects described a relationship between preference and the performance, present in specialist, but lacking in generalists. The lack of the PPH relationship in *A. abdominalis* and *D. rapae* is due to their low host selectivity and their high performance in multiple hosts (in 6 and 5 aphid species, respectively). In *A. ervi*, both its preference and performance were significantly higher in the aphids belonging to the Macrosiphini tribe (as in Zepeda‐Paulo et al., 2013), suggesting a host phylogenetic specialization (Desneux, Blahnik, Delebecque, & Heimpel, 2012). The PPH relationship provides useful clues to classify parasitoids in terms of degree of specialization, although it does not enable strictly separating generalist from oligophagous organisms. Hence, the host specificity index (from Poulin & Mouillot, 2005) provides useful complementary information, quantifying where these species lie on a generalist–specialist continuum. Indeed, when comparing the $S_{\text{TD}}^{\text{*}}$ values of the parasitoids tested in this study and the parasitoids tested in our previous studies, we demonstrated that *A. abdominalis* and *D. rapae* are generalist aphid parasitoids and are able to produce offspring with high prevalence in aphids from both Aphidini and Macrosiphini tribes, whereas *A. ervi* and *L. testaceipes* are moderate specialist aphid parasitoids able producing offspring in aphid species mainly from the Macrosiphini or Aphidini tribe, respectively. Finally, *B. communis* and *B. koreanus* are classified as specialist parasitoids, being able to produce offspring mainly in aphids belonging to the *Aphis* genus (with the exception of *S. graminum*, an aphid species from the Aphidini tribe, still closely related to the *Aphis* genus, Desneux, Barta, Hoelmer, et al., 2009; Desneux, Starý, et al., 2009).

Parasitoid within‐species genetic variability could have some degree of influence on preference‐ and/or performance‐ related traits (Cayetano & Vorburger, 2015; Diehl & Bush, 1984; Raymond, Plantegenest, Gagic, Navasse, & Lavandero, 2015). For example, Derocles et al. (2016) reported that various generalist parasitoid species are composed of biotypes linked to a given host species. This suggests that results on behavioral and/or physiological traits involved in parasitoid specialization might vary slightly according to the actual biotype considered when studying a given parasitoid species. For example, a biotype of *D. rapae* reared on *M. persicae* parasitized only *M. persicae* and *B. brassicae,* whereas another biotype reared on *Hayhurstia atriplicis* was able to parasitize *H. atriplicis*, *M. persicae*, *B. brassicae,* and *A. fabae* (Navasse, Derocles, Plantegenest, & Ralec, 2018). Therefore, examining several biotypes of a same parasitoid species may be useful when characterizing its degree of specialization. It may also help to provide a more accurate assessment when developing biological control programs requiring parasitoids with a high degree of specialization (e.g., classical biological control).

## 5 CONCLUSION

We demonstrated that the preference–performance relationship is present for specialist parasitoids, but not for intermediate specialist–generalist and true generalists, likely owing to combined effects of low selectivity and variable performance in generalist parasitoids (van Klinken, 2000). The generalists are less affected by specific aphid defenses against them (such as endosymbionts, whereas they are strongly affected by general ones that are used against natural enemies (e.g., aphid ability to sequester the toxic compounds). The preference of generalists is not an accurate proxy of actual parasitoid realized host range, i.e., performance. The occurrence (or lack thereof) of such a relationship, as well as the host specificity index, may provide a reliable indicator of actual generalism–specialism in parasitoids.

## CONFLICT OF INTEREST

None declared.

## Data Availability

Data for this study are available at datadryad.org/ (https://doi.org/10.5061/dryad.n88s5r3) (Monticelli et., 2019).

## References

[eva12822-bib-0001] Afsheen, S. , Wang, X. , Li, R. , Zhu, C.‐S. , & Lou, Y.‐G. (2008). Differential attraction of parasitoids in relation to specificity of kairomones from herbivores and their by‐products. Insect Science, 15(5), 381–397. 10.1111/j.1744-7917.2008.00225.x

[eva12822-bib-0002] Alborn, H. T. , Lewis, W. J. , & Tumlinson, J. H. (1995). Host specific recognition kairomone for the parasitoid *Microplitis croceipes* (Cresson*)* . Journal of Chemical Ecology, 21, 1697–1708. 10.1007/BF02033670 24233823

[eva12822-bib-0003] Alikhani, M. , Rezwani, A. , Starý, P. , Kavallieratos, N. G. , & Rakhshani, E. (2013). Aphid parasitoids (Hymenoptera: Braconidae: Aphidiinae) in cultivated and non‐cultivated areas of Markazi Province, Iran. Biologia Section Zoology, 685, 966–973. 10.2478/s11756-013-0234-y

[eva12822-bib-0004] Asplen, M. K. , Bano, N. , Brady, C. M. , Desneux, N. , Hopper, K. R. , Malouines, C. , … Heimpel, G. E. (2014). Specialisation of bacterial endosymbionts that protect aphids from parasitoids. Ecological Entomology, 39(6), 736–739. 10.1111/een.12153

[eva12822-bib-0005] Barbosa, P. (1988). Natural enemies and herbivore–plant interactions: Influence of plant allelochemicals and host specificity In Barbosa P. & Letourneau D. K. (Eds.), Novel aspects of plant interactions (pp. 201–229). New York, NY: Wiley Interscience Publication.

[eva12822-bib-0006] Battaglia, D. , Poppy, G. , Powell, W. , Romano, A. , Tranfaglia, A. , & Pennacchio, F. (2000). Physical and chemical cues influencing the oviposition behaviour of *Aphidius ervi* . Entomologia Experimentalis et Applicata, 94(3), 219–227. 10.1046/j.1570-7458.2000.00623.x

[eva12822-bib-0007] Becker, C. , Desneux, N. , Monticelli, L. , Fernandez, X. , Michel, T. , & Lavoir, A.‐V. (2015). Effects of abiotic factors on HIPV‐mediated interactions between plants and parasitoids. BioMed Research International, 2015, 1–18. 10.1155/2015/342982 PMC4692980 26788501

[eva12822-bib-0008] Bell, W. J. (1991). Searching behaviour. The behavioural ecology of finding resources. London, UK: Chapman and Hall.

[eva12822-bib-0009] Bilodeau, E. , Simon, J.‐C. , Guay, J.‐F. , Turgeon, J. , & Cloutier, C. (2013). Does variation in host plant association and symbiont infection of pea aphid populations induce genetic and behaviour differentiation of its main parasitoid, *Aphidius ervi*? Evolutionary Ecology, 27(1), 165–184. 10.1007/s10682-012-9577-z

[eva12822-bib-0010] Blackman, R. L. , & Eastop, V. F. (2006). Aphids on the world's herbaceous plants and shrubs. Chichester, UK: Wiley.

[eva12822-bib-0011] Bradburne, R. P. , & Mithen, R. 2000 Glucosinolate genetics and the attraction of the aphid parasitoid *Diaeretiella rapae* to Brassica. Proceedings of the Royal Society B: Biological Sciences, 267(1438):89–95. 10.1098/rspb.2000.0971 PMC1690500 10670958

[eva12822-bib-0012] Brodeur, J. , Geervliet, J. B. F. , & Vet, L. E. M. (1998). Effects of *Pieris* host species on life history parameters in a solitary specialist and gregarious generalist parasitoid (*Cotesia* species). Entomologia Experimentalis et Applicata, 86(2), 145–152. 10.1046/j.1570-7458.1998.00275.x

[eva12822-bib-0013] Cayetano, L. , & Vorburger, C. (2015). Symbiont‐conferred protection against Hymenopteran parasitoids in aphids: How general is it? Ecological Entomology, 40(1), 85–93.

[eva12822-bib-0014] Chesnais, Q. , Ameline, A. , Doury, G. , Le Roux, V. , & Couty, A. (2015). Aphid parasitoid mothers don't always know best through the whole host selection process. PLoS ONE, 10(8), 1–16. 10.1371/journal.pone.0135661 PMC4535949 26270046

[eva12822-bib-0015] Coeur d'acier, A. , Jousselin, E. , Martin, J.‐F. , & Rasplus, J.‐Y. (2007). Phylogeny of the genus *Aphis* Linnaeus, 1758 (Homoptera: Aphididae) inferred from mitochondrial DNA sequences. Molecular Phylogenetics and Evolution, 42(3), 598–611. 10.1016/j.ympev.2006.10.006 17113793

[eva12822-bib-0016] Colinet, H. , Salin, C. , Boivin, G. , & Hance, T. (2005). Host age and fitness‐related traits in a koinobiont aphid parasitoid. Ecological Entomology, 30(4), 473–479. 10.1111/j.0307-6946.2005.00716.x

[eva12822-bib-0017] Craig, T. P. , Itami, J. K. , & Price, P. W. (1989). A strong relationship between oviposition preference and larval performance in a shoot‐galling sawfly. Ecology, 70(6), 1691–1699. 10.2307/1938103

[eva12822-bib-0018] De Farias, A. M. I. , & Hopper, K. R. (1999). Oviposition behavior of *Aphelinus asychis* (Hymenoptera: Aphelinidae) and *Aphidius matricariae* (Hymenoptera: Aphidiidae) and defense behavior of their host *Diuraphis noxia* (Homoptera: Aphididae). Environmental Entomology, 28(5), 858–862.

[eva12822-bib-0019] Derocles, S. A. P. , Plantegenest, M. , Rasplus, J.‐Y. , Marie, A. , Evans, D. M. , Lunt, D. H. , & Le Ralec, A. (2016). Are generalist Aphidiinae (Hym. Braconidae) mostly cryptic species complexes? Systematic Entomology, 41(2), 379–391. 10.1111/syen.12160

[eva12822-bib-0020] Desneux, N. , Asplen, M. K. , Brady, C. M. , Heimpel, G. E. , Hopper, K. R. , Luo, C. , Monticelli, L. S. , Oliver, K. M. , & White, J. A. (2018). Intraspecific variation in facultative symbiont infection among native and exotic pest populations: potential implications for biological control. Biological Control, 116, 27–35.

[eva12822-bib-0021] Desneux, N. , Barta, R. J. , Delebecque, C. J. , & Heimpel, G. E. (2009). Transient host paralysis as a means of reducing self‐superparasitism in koinobiont endoparasitoids. Journal of Insect Physiology, 55(4), 321–327. 10.1016/j.jinsphys.2008.12.009 19162033

[eva12822-bib-0022] Desneux, N. , Barta, R. J. , Hoelmer, K. A. , Hopper, K. R. , & Heimpel, G. E. (2009). Multifaceted determinants of host specificity in an aphid parasitoid. Oecologia, 160(2), 387–398. 10.1007/s00442-009-1289-x 19219460

[eva12822-bib-0023] Desneux, N. , Blahnik, R. , Delebecque, C. J. , & Heimpel, G. E. (2012). Host phylogeny and specialisation in parasitoids. Ecology Letters, 15(5), 453–460. 10.1111/j.1461-0248.2012.01754.x 22404869

[eva12822-bib-0024] Desneux, N. , Rabasse, J.‐M. , Ballanger, Y. , & Kaiser, L. (2006). Parasitism of canola aphids in France in autumn. Journal of Pest Science, 79(2), 95–102. 10.1007/s10340-006-0121-1

[eva12822-bib-0025] Desneux, N. , Starý, P. , Delebecque, C. J. , Gariepy, T. D. , Barta, R. J. , Hoelmer, K. A. , & Heimpel, G. E. (2009). Cryptic species of parasitoids attacking the soybean aphid (Hemiptera: Aphididae) in Asia: *Binodoxys communis* and *Binodoxys koreanus* (Hymenoptera: Braconidae: Aphidiinae). Annals of the Entomological Society of America, 102(6), 925–936.

[eva12822-bib-0026] Diehl, S. R. , & Bush, G. L. (1984). An evolutionary and applied perspective of insect biotypes. Annual Review of Entomology, 29(1), 471–504. 10.1146/annurev.en.29.010184.002351

[eva12822-bib-0027] Driessen, G. , Hemerik, L. , & Boonstra, B. (1991). Host selection behaviour of the parasitoid *Leptopilina clavipes*, in relation to survival in hosts. Netherlands Journal of Zoology, 41(2–3), 99–111.

[eva12822-bib-0028] Eben, A. , Benrey, B. , Sivinski, J. , & Aluja, M. (2000). Host species and host plant effects on preference and performance of *Diachasmimorpha longicaudata* (Hymenoptera: Braconidae). Environmental Entomology, 29(1), 87–94.

[eva12822-bib-0029] Felsenstein, J. (1985). Phylogenies and the comparative method. The American Naturalist, 125(1), 1–15. 10.1086/284325

[eva12822-bib-0030] Ferrari, J. , Darby, A. C. , Daniell, T. J. , Godfray, H. C. J. , & Douglas, A. E. (2004). Linking the bacterial community in pea aphids with host‐plant use and natural enemy resistance. Ecological Entomology, 29, 60–65. 10.1111/j.1365-2311.2004.00574.x

[eva12822-bib-0031] Ferrari, J. , & Vavre, F. (2011). Bacterial symbionts in insects or the story of communities affecting communities. Philosophical Transactions of the Royal Society of London. Series B, Biological Sciences, 366(1569), 1389–1400. 10.1098/rstb.2010.0226 21444313 PMC3081568

[eva12822-bib-0032] Francis, F. , Lognay, G. , Wathelet, J.‐P. , & Haubruge, E. (2001). Effects of allelochemicals from first (Brassicaceae) and second (*Myzus persicae* and *Brevicoryne brassicae*) trophic levels on *Adalia bipunctata* . Journal of Chemical Ecology, 27(2), 243–256. 14768813 10.1023/a:1005672220342

[eva12822-bib-0033] Godfray, H. C. J. (1994). Parasitoids: Behavioural and evolutionary ecology. Chichester, UK: Princeton University Press.

[eva12822-bib-0034] Gripenberg, S. , Mayhew, P. J. , Parnell, M. , & Roslin, T. (2010). A meta‐analysis of preference‐performance relationships in phytophagous insects. Ecology Letters, 13(3), 383–393. 10.1111/j.1461-0248.2009.01433.x 20100245

[eva12822-bib-0035] Harvey, P. , & Pagel, M. D. (1991). The comparative method in evolutionary biology. Oxford, UK: Oxford University Press.

[eva12822-bib-0036] Hatano, E. , Kunert, G. , Michaud, J. P. , & Weisser, W. W. (2008). Chemical cues mediating aphid location by natural enemies. European Journal of Entomology, 105(5), 797–806.

[eva12822-bib-0037] Henry, L. M. , Gillespie, D. R. , & Roitberg, B. D. (2005). Does mother really know best? Oviposition preference reduces reproductive performance in the generalist parasitoid *Aphidius ervi* . Entomologia Experimentalis et Applicata, 116(3), 167–174.

[eva12822-bib-0038] Henry, L. M. , Maiden, M. C. , Ferrari, J. , & Godfray, H. C. J. (2015). Insect life history and the evolution of bacterial mutualism. Ecology Letters, 18(6), 516–525. 10.1111/ele.12425 25868533

[eva12822-bib-0039] Honek, A. , Jarosik, V. , Lapchin, L. , & Rabasse, J. (1998). Host choice and offspring sex allocation in the aphid parasitoid *Aphelinus abdominalis* (Hymenoptera: Aphelinidae). Journal of Agricultural Entomology, 15(3), 209–221.

[eva12822-bib-0040] Hopkinson, J. E. , Zalucki, M. P. , & Murray, D. A. H. (2013). Host selection and parasitism behavior of *Lysiphlebus testaceipes*: Role of plant, aphid species and instar. Biological Control, 64(3), 283–290. 10.1016/j.biocontrol.2012.11.016

[eva12822-bib-0041] Hopper, K. R. , Kuhn, K. L. , Lanier, K. , Rhoades, J. H. , Oliver, K. M. , White, J. A. , … Heimpel, G. E. (2018). The defensive aphid symbiont *Hamiltonella defensa* affects host quality differently for *Aphelinus glycinis* versus *Aphelinus atriplicis* . Biological Control, 116, 3–9. 10.1016/j.biocontrol.2017.05.008

[eva12822-bib-0042] Hrček, J. , McLean, A. H. C. , & Godfray, H. C. J. (2016). Symbionts modify interactions between insects and natural enemies in the field. Journal of Animal Ecology, 85(6), 1605–1612. 10.1111/1365-2656.12586 27561159 PMC5082498

[eva12822-bib-0043] Hullé, M. , Turpeau, E. , & Chaubet, B. (2006). Encyclop'aphid, INRA, 10.15454/1.4333379890530916E12

[eva12822-bib-0044] Ives, A. R. , & Godfray, H. C. J. (2006). Phylogenetic analysis of trophic associations. The American Naturalist, 168(1), E1–E14. 10.1086/505157 16874610

[eva12822-bib-0045] Jaenike, J. (1978). On optimal oviposition behavior in phytophagous insects. Theoretical Population Biology, 14(3), 350–356. 10.1016/0040-5809(78)90012-6 751265

[eva12822-bib-0046] Jones, A. M. E. , Bridges, M. , Bones, A. M. , Cole, R. , & Rossiter, J. T. (2001). Purification and characterisation of a non‐plant myrosinase from the cabbage aphid *Brevicoryne brassicae* (L.). Insect Biochemistry and Molecular Biology, 31(1), 1–5. 10.1016/S0965-1748(00)00157-0 11102829

[eva12822-bib-0047] Jones, R. L. , Lewis, W. J. , Bowman, M. C. , Beroza, M. , & Bierl, B. A. (1971). Host seeking stimulants for parasite of corn earworm: Isolation, identification and synthesis. Science, 173(3999), 842–843. 17812196 10.1126/science.173.3999.842

[eva12822-bib-0048] Kavallieratos, N. G. , Tomanovic, Z. , Stary, P. , Athanassiou, C. G. , Sarlis, G. P. , Petrovic, O. , & Niketic, M. A. (2004). A survey of aphid parasitoids (Hymenoptera: Braconidae: Aphidiinae) of Southeastern Europe and their aphid‐plant associations. Applied Entomology and Zoology, 39(3), 527–563. 10.1303/aez.2004.527

[eva12822-bib-0049] Khakasa, S. , Mohamed, S. , Lagat, Z. , Khamis, F. , & Tanga, C. (2016). Host stage preference and performance of the aphid parasitoid *Diaeretiella rapae* (Hymenoptera: Braconidae) on *Brevicoryne brassicae* and *Lipaphis pseudobrassicae* (Hemiptera: Aphididae). International Journal of Tropical Insect Science, 36(1), 10–21. 10.1017/S1742758415000260

[eva12822-bib-0050] VanKlinken, R. D. (2000). Host specificity testing: Why do we do it and how we can do it better In Van Driesche R. G., Heard T. A., McClay A., & Reardon R. (Eds.), Host specificity testing of exotic Arthropod Biological Control Agents – The biological basis for improvement in safety (pp. 54–68). Morgantown, WV: Forest Health Technology Enterprise Team.

[eva12822-bib-0051] Kochetova, N. I. (1978). Factors determining the sex ratio in some entomophagous hymenoptera. Entomological Review, 57, 1–5.

[eva12822-bib-0052] Kos, M. , Houshyani, B. , Achhami, B. B. , Wietsma, R. , Gols, R. , Weldegergis, B. T. , … van Loon, J. J. A. (2012). Herbivore‐mediated effects of glucosinolates on different natural enemies of a specialist aphid. Journal of Chemical Ecology, 38(1), 100–115. 10.1007/s10886-012-0065-2 22258357 PMC3268984

[eva12822-bib-0053] Kouamé, K. L. , & Mackauer, M. (1991). Influence of aphid size, age and behaviour on host choice by the parasitoid wasp *Ephedrus californicus*: A test of host‐size models. Oecologia, 88(2), 197–203. 10.1007/BF00320811 28312132

[eva12822-bib-0054] Kraft, L. J. , Kopco, J. , Harmon, J. P. , & Oliver, K. M. (2017). Aphid symbionts and endogenous resistance traits mediate competition between rival parasitoids. PLoS ONE, 12(7), e0180729. 28700614 10.1371/journal.pone.0180729 PMC5507255

[eva12822-bib-0055] Le Ralec, A. , Anselme, C. , Outreman, Y. , Poirié, M. , Van Baaren, J. , Le Lann, C. C. , & Van Alphen, J. J. M. (2010). Evolutionary ecology of the interactions between aphids and their parasitoids. Comptes Rendus Biologies, 333(6–7), 554–565. 20541166 10.1016/j.crvi.2010.03.010

[eva12822-bib-0056] Li, L. , Miller, D. R. , & Sun, J. (2009). The influence of prior experience on preference and performance of a cryptoparasitoid *Scleroderma guani* (Hymenoptera: Bethylidae) on beetle hosts. Ecological Entomology, 349(6), 725–734.

[eva12822-bib-0057] Luo, C. , Monticelli, L. , Meng, L. , Li, D. , Fan, J. , Zhao, H. , & Hu, Z. (2017). Effect of the endosymbiont *Regiella insecticola* on an aphid parasitoid. Entomologia Generalis, 36(4), 300–307. 10.1127/entomologia/2017/0443

[eva12822-bib-0058] Mackauer, M. (1986). Growth and developmental interactions in some aphids and their hymenopterous parasites. Journal of Insect Physiology, 32(4), 275–280. 10.1016/0022-1910(86)90039-9

[eva12822-bib-0059] Mackauer, M. , Michaud, M. R. , & Völkl, W. (1996). Host choice by aphidiid parasitoids (Hymenoptera: Aphidiidae): Host recognition, host quality, and value. The Canadian Entomologist, 128(6), 959–980.

[eva12822-bib-0060] McCormick Clavijo, A. , Unsicker, S. B. , & Gershenzon, J. (2012). The specificity of herbivore‐induced plant volatiles in attracting herbivore enemies. Trends in Plant Science, 17(5), 303–310. 10.1016/j.tplants.2012.03.012 22503606

[eva12822-bib-0061] McLean, A. H. C. , & Godfray, H. C. J. (2015). Evidence for specificity in symbiont‐conferred protection against parasitoids. Proceedings of the Royal Society B: Biological Sciences, 282(1811), 20150977 10.1098/rspb.2015.0977 PMC4528558 26136451

[eva12822-bib-0062] Mclean, A. H. C. , Hrček, J. , Parker, B. J. , & Godfray, H. C. J. (2017). Cascading effects of herbivore protective symbionts on hyperparasitoids. Ecological Entomology, 42(5), 601–609. 10.1111/een.12424

[eva12822-bib-0063] Michaud, J. P. , & Mackauer, M. (1994). The use of visual cues in host evaluation by aphidiid wasps: I. Comparison between three *Aphidius* parasitoids of the pea aphid. Entomologia Experimentalis et Applicata, 70(3), 273–283. 10.1111/j.1570-7458.1994.tb00756.x

[eva12822-bib-0064] Michaud, J. P. , & Mackauer, M. (1995). The use of visual cues in host evaluation by aphidiid wasps: II. Entomologia Experimentalis et Applicata, 74(3), 267–275. 10.1111/j.1570-7458.1995.tb01900.x

[eva12822-bib-0065] Monticelli, L. S. (2018). Study of ecological factors modulating parasitoid host range. PhD, Doctoral school of the Université Nice Côte d'Azur, 335p.

[eva12822-bib-1066] Monticelli, L. S. , Nguyen, L. T. H. , Amiens‐Desneux, E. , Luo, C. , Lavoir, A. , Gatti, J. & Desneux, N. (2019). Data from: The preference‐performance relationship as a means of classifying parasitoids according to their specialization degree. Dryad Digital Repository. 10.5061/dryad.n88s5r3. PMC6708433 31462919

[eva12822-bib-0066] Monticelli, L. S. , Outreman, Y. , Frago, E. , & Desneux, N. (2019). Impact of host endosymbionts on parasitoid host range – From mechanisms to communities. Current Opinion in Insect Science, 32, 77–82. 10.1016/j.cois.2018.11.005 31113635

[eva12822-bib-0067] Mooney, K. , Jones, P. , & Agrawal, A. (2008). Coexisting congeners: Demography, competition, and interactions with cardenolides for two milkweed‐feeding aphids. Oikos, 117(3), 450–458. 10.1111/j.2007.0030-1299.16284.x

[eva12822-bib-0068] Navasse, Y. , Derocles, S. A. P. , Plantegenest, M. , & Le Ralec, A. (2018). Ecological specialization in *Diaeretiella rapae* (Hymenoptera: Braconidae: Aphidiinae) on aphid species from wild and cultivated plants. Bulletin of Entomological Research, 108(02), 175–184. 28770687 10.1017/S0007485317000657

[eva12822-bib-0069] Nguyen, L.‐T.‐H. , Monticelli, L. S. , Desneux, N. , Metay‐Merrien, C. , Amiens‐Desneux, E. , & Lavoir, A.‐V. (2018). Bottom‐up effect of water stress on the aphid parasitoid *Aphidius ervi* . Entomologia Generalis, 38(1), 15–27. 10.1127/entomologia/2018/0575

[eva12822-bib-0070] Nylin, S. , & Janz, N. (1993). Oviposition preference and larval performance in *Polygonia calbum* (Lepidoptera: Nymphalidae): The choice between bad and worse. Ecological Entomology, 18(4), 394–398. 10.1111/j.1365-2311.1993.tb01116.x

[eva12822-bib-0071] Oliver, K. M. , Degnan, P. H. , Hunter, M. S. , & Moran, N. A. (2009). Bacteriophages encode factors required for protection in a symbiotic mutualism. Science, 325(5943), 992–994. 10.1126/science.1174463 19696350 PMC5473335

[eva12822-bib-0072] Oliver, K. M. , Moran, N. A. , & Hunter, M. S. (2005). Variation in resistance to parasitism in aphids is due to symbionts not host genotype. Proceedings of the National Academy of Sciences of the United States of America, 102(36), 12795–12800. 10.1073/pnas.0506131102 16120675 PMC1200300

[eva12822-bib-0073] Oliver, K. M. , Russell, J. A. , Moran, N. A. , & Hunter, M. S. (2003). Facultative bacterial symbionts in aphids confer resistance to parasitic wasps. Proceedings of the National Academy of Sciences of the United States of America, 100(4), 1803–1807. 10.1073/pnas.0335320100 12563031 PMC149914

[eva12822-bib-0074] Omkar, & Mishra, G. (2005). Preference–performance of a generalist predatory ladybird: A laboratory study. Biological Control, 34(2), 187–195. 10.1016/j.biocontrol.2005.05.007

[eva12822-bib-0075] Parker, B. J. , Spragg, C. J. , Altincicek, B. , & Gerardo, N. M. (2013). Symbiont‐mediated protection against fungal pathogens in pea aphids: A role for pathogen specificity? Applied and Environmental Microbiology, 79(7), 2455–2458. 10.1128/AEM.03193-12 23354709 PMC3623210

[eva12822-bib-0076] Pons, X. , Lumbierres, B. , Antoni, R. , & Stary, P. (2011). Parasitoid complex of alfalfa aphids in an IPM intensive crop system in northern Catalonia. Journal of Pest Science, 84(4), 437–445. 10.1007/s10340-011-0383-0

[eva12822-bib-0077] Poulin, R. , & Mouillot, D. (2003). Parasite specialization from a phylogenetic perspective: A new index of host specificity. Parasitology, 126(5), 473–480. 10.1017/S0031182003002993 12793652

[eva12822-bib-0078] Poulin, R. , & Mouillot, D. (2005). Combining phylogenetic and ecological information into a new index of host specificity. Journal of Parasitology, 91(3), 511–514. 10.1645/GE-398R 16108540

[eva12822-bib-0079] Pratt, C. , Pope, T. W. , Powell, G. , & Rossiter, J. T. (2008). Accumulation of glucosinolates by the cabbage aphid *Brevicoryne brassicae* as a defense against two coccinellid species. Journal of Chemical Ecology, 34(3), 323–329. 10.1007/s10886-007-9421-z 18270780

[eva12822-bib-0080] R Core Team . (2017). R: A language and environment for statistical computing. Vienna, Austria: R Foundation for Statistical Computing Retrieved from https://www.R-project.org/

[eva12822-bib-0081] Raymond, L. , Plantegenest, M. , Gagic, V. , Navasse, Y. , & Lavandero, B. (2015). Aphid parasitoid generalism: Development, assessment, and implications for biocontrol. Journal of Pest Science, 89(1), 7–20.

[eva12822-bib-0082] Sadeghi, H. , & Gilbert, F. (1999). Individual variation in oviposition preference, and its interaction with larval performance in an insect predator. Oecologia, 118(4), 405–411. 10.1007/s004420050742 28307407

[eva12822-bib-0083] Starý, P. (1993). The fate of released parasitoids (Hymenoptera: Braconidae, Aphidiinae) for biological control of aphids in Chile. Bulletin of Entomological Research, 83(4), 633–639. 10.1017/S0007485300040062

[eva12822-bib-0084] Thompson, J. N. (1988). Evolutionary ecology of the relationship between oviposition preference and performance of offspring in phytophagous insects. Entomologia Experimentalis et Applicata, 47(1), 3–14. 10.1111/j.1570-7458.1988.tb02275.x

[eva12822-bib-0085] Toft, S. , & Wise, D. H. (1999). Growth, development, and survival of a generalist predator fed single‐ and mixed‐species diets of different quality. Oecologia, 119(2), 191–197. 10.1007/s004420050776 28307968

[eva12822-bib-0086] Vet, L. E. M. (1985). Olfactory microhabitat location in some Eucoilid and Alysiine species (Hymenoptera), larval parasitoid of Diptera. Netherlands Journal of Zoology, 35(4), 720–730.

[eva12822-bib-0087] Vet, L. E. M. , & Dicke, M. (1992). Ecology of infochemical use by natural enemies in a tritrophic context. Annual Review of Entomology, 37(1), 141–172. 10.1146/annurev.en.37.010192.001041

[eva12822-bib-0088] Vinson, S. B. (1985). The behaviour of parasitoids In Kerkut G. A. & Gilbert L. I. (Eds.), Comprehensive insect physiology, biochemistry and pharmacology (Vol. 9, pp. 417–469). Oxford, UK: Pergamon Press.

[eva12822-bib-0089] Völkl, W. , & Mackauer, M. (2000). Oviposition behaviour of aphidiine wasps (hymenoptera: Braconidae, aphidiinae): Morphological adaptations and evolutionary trends. The Canadian Entomologist, 132(02), 197–212. 10.4039/Ent132197-2

[eva12822-bib-0090] von Burg, S. , Ferrari, J. , Muller, C. B. , & Vorburger, C. (2008). Genetic variation and covariation of susceptibility to parasitoids in the aphid *Myzus persicae*: No evidence for trade‐offs. Proceedings of the Royal Society B: Biological Sciences, 275(1638), 1089–1094. 10.1098/rspb.2008.0018 PMC2600915 18270153

[eva12822-bib-0091] von Dohlen, C. D. , Rowe, C. A. , & Heie, O. E. (2006). A test of morphological hypotheses for tribal and subtribal relationships of Aphidinae (Insecta: Hemiptera: Aphididae) using DNA sequences. Molecular Phylogenetics and Evolution, 38(2), 316–329. 10.1016/j.ympev.2005.04.035 16368250

[eva12822-bib-0092] Vorburger, C. , Gehrer, L. , & Rodriguez, P. (2009). A strain of the bacterial symbiont *Regiella insecticola* protects aphids against parasitoids. Biology Letters, 6(1), 109–111. 19776066 10.1098/rsbl.2009.0642 PMC2817266

[eva12822-bib-0093] Wahab, W. (1985). Observations on the biology and behaviour of *Aphelinus abdominalis* Dalm. (Hym., Aphelinidae), a parasite of aphids. Zeitschrift für Angewandte Entomologie, 100(3), 290–296.

[eva12822-bib-0094] Weber, G. (1986). Ecological genetics of host plant exploitation in the green peach aphid, *Myzus persicae* . Entomologia Experimentalis et Applicata, 40(2), 161–168. 10.1111/j.1570-7458.1986.tb00498.x

[eva12822-bib-0095] Wyckhuys, K. A. G. , Stone, L. , Desneux, N. , Hoelmer, K. A. , Hopper, K. R. , & Heimpel, G. E. (2008). Parasitism of the soybean aphid, *Aphis glycines* by *Binodoxys communis*: The role of aphid defensive behaviour and parasitoid reproductive performance. Bulletin of Entomological Research, 98(4), 361–370. 18294416 10.1017/S000748530800566X PMC2670187

[eva12822-bib-0096] Zepeda‐Paulo, F. A. , Ortiz‐Martínez, S. A. , Figueroa, C. C. , & Lavandero, B. (2013). Adaptive evolution of a generalist parasitoid: Implications for the effectiveness of biological control agents. Evolutionary Applications, 6(6), 983–999. 10.1111/eva.12081 24062806 PMC3779098

