# Supplementary material for: The preference–performance relationship as a means of classifying parasitoids according to their specialization degree
Source: Evol Appl. 2019 Jun 17;12(8):1626–40. doi: 10.1111/eva.12822 (PMC6708433; doi:10.1111/eva.12822)
Supplement: Supplementary file 1 [file EVA-12-1626-s001.docx]

**SUPPLEMENTARY TABLE AND FIGURE.**

**Supplementary materials and methods 1.**

*DNA extraction.* Each sampling was washed in 70 % ethanol for 2 to 5 minutes (aphid size dependent), rinsed in PBS (phosphate buffer solution) for 1 minute and finally washed in pure water. Samples were homogenized with piston (1 piston/sample) in Lysis buffer for DNA extraction. Then, samples were placed in 10 µL of RNase A, 50 µL of lysozyme and 20 µL of protease K and incubated at 55 °C for 3 hours. For DNA purification, samples were centrifuged at 3000 rpm for 5 minutes and the supernatants were collected. 1mL of absolute ethanol and 50 µL of sodium acetate were added and the mixture was placed at -20°C overnight. Samples were then centrifuged at 14000 rpm for 20 minutes and the supernatants were removed. After adding 1mL of 70 % ethanol, the mixture was homogenized and centrifuged at 14000 rpm for 5 minutes. After 5 minutes at room temperature to let the pellet dry, 50 µL of pure water was added to the pellet and then stored at -20 °C until used. The quantity and quality of the DNA were measured using NanoDrop and diluted to obtain 50 ng of DNA /µL for each sample.

*PCR amplification.* Diagnostic PCR reactions with symbiont-specific primer pairs (Sup table 1) were conducted in 1.5 % agarose gel stained with ethidium bromide and visualized with UV light to test the presence of facultative symbionts. Each symbiont was tested with a positive control (DNA from aphid species) and two negative controls (two pea aphid genotypes that were known to harbor only the primary endosymbiont *Buchnera aphidicola*). Furthermore, the quality of the extraction was tested by PCR on the primary endosymbiont: *B. aphidicola*. Finally, bands showing a signal were removed from the gel, purified (with Min Elute PCR purification kit) and sequenced to check the symbiont identity (validated when the sequence was at least 95% similar).

**Supplementary table T1.**

Endosymbiont targeted, the target gene and the primer name used to detect the symbiont present in the different aphid species.

| **Target** | **Target gene** | **Primer name** | **Sequences** |
| --- | --- | --- | --- |
| *Hamiltonella defensa* (T-type) | *16S rDNA* | 16S-8F  16S-480R | AGAGTTTGATCMTGGCTCAG  GGTATTCGCATTTATCGCTTC |
| *Buchnera* | *DnaK* | BuchDnaK_12F  BuchDnaK_162R | TATTGGTATTGACTTGGGAA  AGCAGGTTGTCCTACTAAAAC |
| *Regiella insecticola* (U-type) | *16S rDNA* | 16S-8F  16S-R2 | AGAGTTTGATCMTGGCTCAG  TCGGACGCCATAACACTAGG |
| *Rickettsiella* | *16S rDNA* | P136F-16S  P136Ric-470R-16S | GGGCCTTGCGCTCTAGGT  TGGGTACCGTCACAGTAATCGA |
| PAXS | 16S rDNA | PAXS F-16S  PAXS R-16S | AGTTTGATCATGGCTCAGATTG  GCAACACTCTTTGCATTGCT |
| *Serratia symbiotica* (R-type) | *16S rDNA* | 16S-8F  PASS1140R | AGAGTTTGATCMTGGCTCAG  TTTGAGTTCCCGACTTTATCG |
| *Rickettsia* | *16S rDNA* | ricCsA-318-F-GltA  ricCsA-318-R-GltA | TGATCATGAGCAAAATGCTT  TCTAGCTGCCCAAGTTCTTT |
| *Spiroplasma* | *16S rDNA* | Spi 618834-F-16S  Spi 618834-R | GTGGCAAGCGTTATCCGGAT  CCCACGCTTTCGTGCCACAA |
| *Wolbachia* | *FtsZ* | Wol-FtsZ-F  Wol-FtsZ-R | TTGCAGAGCTTGGACTTGAA  CATATCTCCGCCACCAGTAA |
| *Arsenophonus* | *yaeT* | Ars-yaeT-F  Ars-yaeT-R | GCATACGGTTCAGACGGGTTTG  GCCGAAACGCCTTCAGAAAAG |

**Supplementary table T2.** Proportion of aphids detected, accepted, and stung by parasitoids as well as the proportion of egg, larvae, mummy and adult *Aphelinus abdominalis*, *Aphidius ervi* and *Diaeretiella rapae* upon the encounter of different host plant species.

|  | **Host plants** | **Parasitoid preference** | | | **Parasitoid performance** | | |  |
| --- | --- | --- | --- | --- | --- | --- | --- | --- |
|  |  | **% Detected ^a^** | **% Accepted** | **% Stung** | **% Larvae** | **% Mummy** | **% Adult** | |
| ***Aphelinus abdominalis*** | Potato | 0.97 | 0.94 b | 0.88 a | 0.92 b | 0.98 | 0.90 | |
|  | Tomato | 1.00 | 1.00 a | 0.88 a | 1.00 a | 0.94 | 0.85 | |
|  | Bean | 0.98 | 0.59 c | 0.40 b | 0.90 b | 0.86 | 0.78 | |
|  | Wheat | 0.99 | 0.83 b | 0.67 a | 0.85 b | 0.66 | 0.59 | |
|  | Cabbage | 0.97 | 0.75 b | 0.60 a | 0.81 b | 0.53 | 0.49 | |
|  | Asclepias | 1.00 | 0.89 b | 0.75 a | 0.05 c | 0.00 | 0.00 | |
|  | Squash | 0.91 | 0.34 c | 0.25 b | 0.00 c | 0.00 | 0.00 | |
| ***Aphidius ervi*** | Tomato | 1.00 | 0.88 | 0.48 | 0.53 | 0.52 | 0.43 | |
|  | Potato | 1.00 | 0.96 | 0.86 | 0.48 | 0.47 | 0.29 | |
|  | Bean | 0.97 | 0.64 | 0.49 | 0.61 | 0.32 | 0.25 | |
|  | Wheat | 0.95 | 0.76 | 0.45 | 0.47 | 0.20 | 0.15 | |
|  | Squash | 0.96 | 0.77 | 0.55 | 0.67 | 0.23 | 0.09 | |
|  | Cabbage | 0.98 | 0.81 | 0.45 | 0.54 | 0.35 | 0.07 | |
|  | Asclepias | 0.98 | 0.68 | 0.47 | 0.67 | 0.20 | 0.07 | |
| ***Diaeretiella rapae*** | Cabbage | 0.98 | 0.80 | 0.63 | 0.93 a | 0.66 a | 0.57 a | |
|  | Squash | 0.93 | 0.47 | 0.24 | 0.86 a | 0.63 a | 0.38 a | |
|  | Wheat | 0.92 | 0.62 | 0.45 | 0.84 a | 0.39 a | 0.34 a | |
|  | Asclepias | 0.97 | 0.74 | 0.34 | 0.83 a | 0.03 b | 0.00 b | |
|  | Potato | 0.77 | 0.43 | 0.14 | 0.00 b | 0.00 b | 0.00 b | |
|  | Tomato | 0.93 | 0.37 | 0.17 | 0.00 b | 0.00 b | 0.00 b | |
|  | Bean | 0.80 | 0.44 | 0.31 | 0.00 b | 0.00 b | 0.00 b | |

For each parasitoid species, proportions followed by the same letter are not significantly different (GLMs followed by a multi comparison test).
^a^ No significant difference for each parasitoid species tested.

**Supplementary figure S1.** Relationship between the stinging rate (preference) and the emergence rate (performance) of three generalist aphid parasitoids (*Aphelinus abdominalis* (A)*, Aphidius ervi* (B) and *Diaeretiella rapae* (C) when encountered twelve different aphid species*.*

**Figure S1. A.** *Aphelinus abdominalis*

**Figure S1. B.** *Aphidius ervi*

**Figure S1. C.** *Diaeretiella rapae*
